# Artificial Neural Network Modeling of a CMOS Differential Low-Noise Amplifier Using the Bayesian Regularization Algorithm

**DOI:** 10.3390/s23218790

**Published:** 2023-10-28

**Authors:** Bhuvaneshwari Subburaman, Vignesh Thangaraj, Vadivel Balu, Uma Maheswari Pandyan, Jayshri Kulkarni

**Affiliations:** 1 ECE Department, Mangayarkarasi College of Engineering, Madurai 625402, Tamil Nadu, India; sp.bhuvana@gmail.com (B.S.); vignesh.thangaraj85@gmail.com (V.T.); vadivelbalu.eee@gmail.com (V.B.); 2 ECE Department, Velammal College of Engineering and Technology, Madurai 625009, Tamil Nadu, India; umamahes.p@gmail.com; 3 Department of Electrical and Computer Engineering, Baylor University, Waco, TX 76798, USA

**Keywords:** gain boosting, differential cascode, capacitor cross-coupling, low-noise amplifier, ANN, Bayesian regularization

## Abstract

The purpose of this communication is to present the modeling of an Artificial Neural Network (ANN) for a differential Complementary Metal Oxide Semiconductor (CMOS) Low-Noise Amplifier (LNA) designed for wireless applications. For satellite transponder applications employing differential LNAs, various techniques, such as gain boosting, linearity improvement, and body bias, have been individually documented in the literature. The proposed LNA combines all three of these techniques differentially, aiming to achieve a high gain, a low noise figure, excellent linearity, and reduced power consumption. Under simulation conditions at 5 GHz using Cadence, the proposed LNA demonstrates a high gain (S21) of 29.5 dB and a low noise figure (NF) of 1.2 dB, with a reduced supply voltage of only 0.9 V. Additionally, it exhibits a reflection coefficient (S11) of less than −10 dB, a power dissipation (Pdc) of 19.3 mW, and a third-order input intercept point (IIP3) of 0.2 dBm. The performance results of the proposed LNA, combining all three techniques, outperform those of LNAs employing only two of the above techniques. The proposed LNA is modeled using PatternNet BR, and the simulation results closely align with the results of the developed ANN. In comparison to the Cadence simulation method, the proposed approach also offers accurate circuit solutions.

## 1. Introduction

Significant progress has been made in the realm of wireless communication systems thanks to the development of fully integrated Complementary Metal Oxide Semiconductor (CMOS) receiver front-ends. In satellite communication systems, a satellite transponder comprises a series of interconnected elements that establish a communication link between the transmitting and receiving antennas. Among these elements are the Band-Pass Filter (BPF), Low-Noise Amplifier (LNA), mixer, and power amplifier. The LNA serves as a fundamental component and plays a pivotal role as the initial block in satellite transponders. It is widely recognized that designing this first block poses a considerable challenge, as its performance profoundly influences the subsequent stages in terms of both the selectivity and sensitivity of the receiver.

To meet the high sensitivity demands, pioneering studies are documented in References [1,2,3,4,5,6,7,8,9,10,11,12,13,14,15,16,17,18,19,20,21,22,23,24,25,26,27,28,29,30,31,32,33,34,35,36]. For instance, Tae-Sung Kim et al. presented an LNA operating at 2 GHz in [1], employing post-linearization techniques with 0.18 µm CMOS technology. However, the performance of the receiver is constrained by the noise factor primarily influenced by the LNA. Hence, achieving a high sensitivity necessitates an LNA with a substantial gain, minimal noise figure, and low power consumption. Given the LNA discussed in [1], the implementation of the cascode topology for LNA design is detailed in [2]. This topology offers significant advantages, including a high gain, a low noise figure, a broader bandwidth, low power consumption, excellent reverse isolation, and stability. However, it is worth noting that the reported LNA is susceptible to parasitic inductance.

The widely recognized differential topology is discussed in [3,4,5,6,7,8,9,10]. The primary advantage of this configuration lies in its ability to reject common-mode noise and its reduced sensitivity to substrate and supply noise. This topology is also well suited for connection to a double-balanced mixer [3], which is typically the subsequent component in the receiver chain. Furthermore, this topology demonstrates the lowest noise figure among the reported designs [4], along with an impressive third-order input intercept point and a 1 dB compression point. It is worth noting, however, that the power consumption is slightly higher than in other reported topologies.

It is essential to emphasize that, since satellites rely on solar cells, achieving optimal performance with low power consumption is imperative to conserve battery life, particularly under very low supply voltages. Given this perspective and the aim of minimizing power usage, various techniques have been explored in the current state of the art. The current-reuse (CR) technique, as documented in [11,12], enables the sharing of the DC current among transistors, thereby reducing power consumption without compromising gain. However, it is important to note that this technique is the most advantageous for applications requiring higher voltage. Conversely, the complementary current-reuse technique, as described in [13], is well-suited for achieving low power while also catering to applications with lower voltage requirements. This approach involves the use of complementary transistors that distribute the current between the input and output stages.

Significantly, the adoption of this technique has implications for gain values. Additionally, the sub-threshold biasing technique, as documented in [14,15,16], has been employed to achieve exceedingly low power consumption (in the µW range). In this method, the MOS transistor operates in the weak inversion region, which necessitates the use of oversized transistors to enhance gain. Currently, wireless devices are being scaled down to achieve miniaturization, thanks to advancements in CMOS technology. Consequently, a low-voltage supply technique is discussed in References [17,18], primarily catering to applications that require less than 1V to operate. However, it is important to note that the use of this technique leads to reduced linearity. Furthermore, the forward body biasing (FBB) technique, detailed in [19,20], merits consideration. While this technique does contribute to a lower threshold voltage without compromising the gain, noise figure, and supply voltage, it is worth acknowledging that maintaining linearity remains a challenging task for researchers and LNA designers.

It is important to acknowledge that the primary source of nonlinearity in an MOS transistor resides in the transconductance available at the input stage. To attain superior linearity, various techniques for improving linearity, as discussed in [21], are also explored. One such approach is the utilization of an auxiliary path, commonly known as the feedforward technique, as described in [22]. This technique is employed to nullify the third-order harmonic at the primary output, which is crucial for achieving enhanced LNA performance. However, it comes at the cost of increased power dissipation and a reduction in gain. In [23], a low-impedance LC trap network is implemented to resonate the LNA in conjunction with the nonlinear signals at the input, effectively canceling them out. Notably, this technique is well-suited for MOSFET transistors when operating outside the strong inversion region.

In [24], an optimal biasing technique is applied at the input side to generate a current or voltage with the aim of mitigating the third-order nonlinearity component (gm_3_). However, it is important to note that this approach is sensitive to variations in bias. However, the injection of a second-order inter-modulation (IM_2_) technique, as introduced in [25], proves to be effective in suppressing third-order inter-modulation distortion (IM_3_) without the need for an auxiliary path. This results in improved linearity without compromising noise, gain, and power consumption. Nonetheless, it may lead to a differential output from the main path and a potential degradation of IIP_2_.The multiple-gated transistor technique, detailed in [26,27,28,29], involves the use of two transistors operating in both the strong and weak inversion regions.

In this context, the negative peak of the second-order nonlinear term, which contributes to the IM_3_ of the main transistor, is offset by the positive peak of the auxiliary transistor. However, this approach still encounters second-order distortion combined with harmonic feedback. An alternative method is the modified derivative superposition (DS) technique, as detailed in References [30,31,32,33], employing two transistors akin to those in [26] and two source inductors. This circuit allows for the direct tuning of third-order inter-modulation distortion from the input stage, albeit at the expense of reduced gain and noise performance. The IMD sinker technique, presented in [34], utilizes an NMOS diode connected in parallel to a resistor–capacitor (RC) circuit. Notably, this technique can partially mitigate IMD_3_ at the input without impacting the gain and noise figure. Lastly, the dual capacitive cross-coupling (CCC) technique, as reported in [35], combines active and passive CCC to enhance loop gain and linearity. However, due to the larger passive CCC, there is a slight degradation in the input matching bandwidth.

Upon a thorough investigation of the recently reported state-of-the-art advancements, it becomes evident that designing an LNA to attain superior performance, including a high gain, low power consumption, a minimal noise figure, space efficiency, and cost-effectiveness, presents a set of formidable challenges for both researchers and LNA designers. This challenge arises from the fact that satellite transponders receive weak signals with inherent noise, caused by weather conditions and interference from Earth stations. Consequently, there is a pressing need to bolster these feeble signals with a combination of a high gain, exceptionally low noise, reduced nonlinearity, and low power consumption to prepare them for further processing. Such objectives are made achievable through the proposed LNA, which leverages a combination of techniques, including current reuse for gain improvement, body biasing for low power consumption, and capacitor cross-coupling for enhancing linearity in the C band, tailored specifically for satellite transponders.

Meeting the desired requirements in LNA design for RF frequencies is a highly challenging task, primarily due to the numerous non-ideal factors encountered. This challenge is compounded by the use of electromagnetic solvers, such as Cadence, ADS, and HFSS, which rely on intricate analytical and mathematical models [37]. An integral part of the design process involves repetitive trial-and-error experiments, demanding a significant investment of time, extensive memory resources, and specialized equipment. The complexity further intensifies when numerous simulations must be repeated for various circuit parameters to optimize the RF performance of the LNA. Therefore, there is a need for a more efficient technique that consumes fewer resources and possesses the capability to model LNAs comprehensively, approximating all design parameters. Artificial Neural Networks (ANNs) stand out as a modeling technique with the capacity to learn from data, approximate unseen data, and model nonlinear parameters [38]. In this work, we propose the optimization of LNA parameters for various CMOS LNAs, including a single-ended LNA, a differential LNA, and a current-reuse LNA, utilizing MLPANN. However, it is essential to note that only two output parameters, the gain and noise figure (NF), are considered in this study.

ANN is employed through both a direct and an inverse approach to model the interdependence of performance parameters and circuit parameters. In the direct approach, ANN is employed to model LNA output performance parameters with respect to input circuit parameters. Conversely, in the inverse approach, ANN is utilized to derive circuit parameters based on the output performance parameters. It is worth noting that the recent literature has explored the application of ANN in modeling LNAs.

In [39], an ANN model was introduced to address LNA impedance matching using the Smith chart. However, a complete ANN model for the entire LNA is still pending development. Another ANN model related to LNA was presented in [40], offering a comparison of accuracy and scalability among various metamodeling methods for the challenging task of modeling an entire LNA RF circuit block. This study employed the Bayesian regularization (BR) algorithm. Nevertheless, there is room for improvement in the selection of orders for the rational function, and further theoretical investigation of SVM is required to understand the significant disparity with ANN. In [41], the modeling of CMOS LNA was achieved using the Adaptive Neuro-Fuzzy Inference System (ANFIS), demonstrating significantly lower errors than models developed using MLP and RBF. A different approach was described in [42], which outlined a method for RF LNA circuit synthesis determining parameter values through a set of ANNs aided by a Genetic Algorithm (GA). It is worth noting that GA required a relatively high number of generations. In [43], the modeling of an LNA was proposed using the Levenberg–Marquardt (LM) algorithm with a limited set of circuit parameters, such as frequency (f), drain-to-source voltage (Vds), drain-to-source current (Ids), and temperature (T). However, matching networks were not part of the model. Another approach was suggested in [44], focusing on solving highly nonlinear design problems for LNAs and Reflect Array Antennas (RAs) using ANN in conjunction with metaheuristic search algorithms. The results indicated successful minimization of the cost function, validated through 3D EM simulators for microwave circuits. In [45], an LNA was modeled using the LM algorithm, employing frequency (f), drain-to-source voltage (Vds), drain-to-source current (Ids), and temperature (T) as input parameters. Nevertheless, the modeling of matching networks was not included. From the review of the available literature, it is evident that both LM and BR algorithms have been applied for LNA modeling and synthesis. In [46], the proposal centered on surrogate modeling for a tunable LNA spanning a range from 2 to 3 GHz. Three- and six-dimensional surrogate models were constructed to illustrate the impact of bond wires on various LNA design metrics. Building upon these prior developments, this work endeavors to predict the different parameters of CMOS LNAs based on collected simulation data.

The objective of this work is to model a CMOS LNA using the Bayesian regularization algorithm, known for its robustness and resistance to overtraining and over-fitting. This work primarily focuses on the ANN modeling of a CMOS differential LNA using a direct approach. This paper presents the ANN modeling of a differential LNA using the BR algorithm, which incorporates techniques like current reuse (CR) for gain enhancement, capacitor cross-coupling (CCC) for improving linearity, and body biasing (BB) for low power consumption. The performance results of the proposed LNA, which combines all three techniques, surpass those of previously reported LNAs. Furthermore, the neural network modeling results closely align with the simulation results obtained from Cadence. The novelty of this work lies in the integration of various techniques to achieve optimized performance in multiple parameters, including gain, the noise figure (NF), power dissipation, and linearity, simultaneously with computer-aided modeling through ANN.

This paper is organized as follows: Section 2 discusses the CMOS differential LNA and its impact on LNA gain and linearity due to the proposed techniques. Section 3 covers the neural network model, Section 4 outlines the ANN modeling of the proposed LNA, Section 5 provides a brief overview of the results and discussion, and, finally, Section 6 concludes the paper.

## 2. CMOS Differential LNA

**Figure 1:**
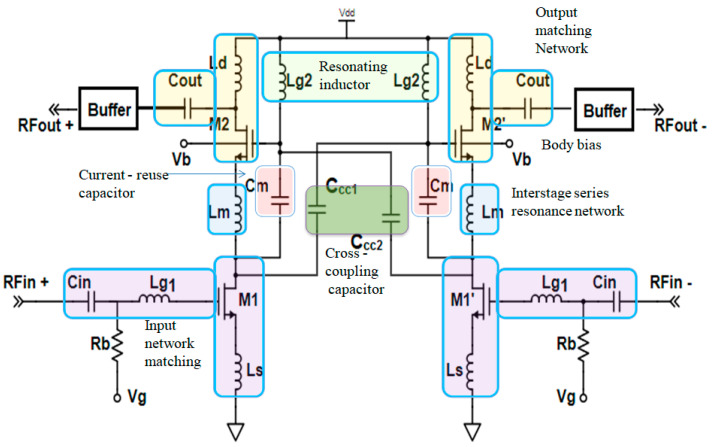
Schematic of proposed differential LNA.

A schematic of the proposed CMOS differential LNA designed for C-band operation is illustrated in Figure 1. As evident in Figure 1, the proposed LNA comprises three stages: the input and output matching network, the interstage series resonance network, and buffers. The inductors (L_s_ and L_g1_) in the common-source (CS) stage are meticulously designed to achieve input impedance matching and ensure low-noise performance within the C band. The aspect ratios of MOS transistors, namely, M_1_ and M_2_, are chosen to achieve appropriate input and output matching. To guarantee the correct operation of the transistors, a biasing circuit is devised to establish the proper DC operating point. This involves configuring the bias current and voltage levels to maintain the transistors within their active region while minimizing power consumption. In LNAs, noise analysis and optimization are of paramount importance. To conduct a comprehensive noise analysis, techniques such as noise matching and impedance matching are employed to minimize the noise figure effectively.

The interstage series resonance inductor (L_m_) serves to eliminate the impact of parasitic capacitance between the common-source (CS) transistor M_1_ and the cascode transistor M_2_, thereby enhancing power transfer efficiency. The current-reuse capacitor (C_m_) plays a crucial role in augmenting the transconductance (g_m2_) of the cascode device by enabling the sharing of the input current with the output device.

The cascode transistor operates in a common-gate (CG) configuration, specifically designed to mitigate the impact of parasitic gate–drain capacitance in the common-source (CS) stage. This configuration serves to increase the output impedance and enhance input/output isolation. The inclusion of a drain inductor (L_d_) and an output capacitor (Cout) in the cascode stage is carefully engineered to achieve the desired output matching.

To strike a balance between bandwidth and gain, several aspects are addressed, including the transistor sizes, input/output matching networks, the addition of a buffer at the output side, and the incorporation of feedback components. These measures are meticulously executed to attain the desired gain and bandwidth characteristics while ensuring system stability.

To enhance linearity, cross-coupling capacitors C_CC1_ and C_CC2_ are incorporated. These capacitors resonate with the gate inductor (L_g2_), effectively canceling out the nonlinearity associated with the common-source (CS) transistor (M_1_).

To further optimize performance, body biasing is employed by connecting the supply voltage (V_b_) to the bulk terminal of cascode transistor M_2_. This adjustment reduces the threshold of M_2_, preventing it from entering the weak inversion region and, consequently, reducing power consumption, especially at lower supply voltages.

The designed LNA underwent extensive simulations using EDA tools to validate its performance within the C band.

### 2.1. Small-Signal Equivalent of the Proposed Differential LNA

**Figure 2:**
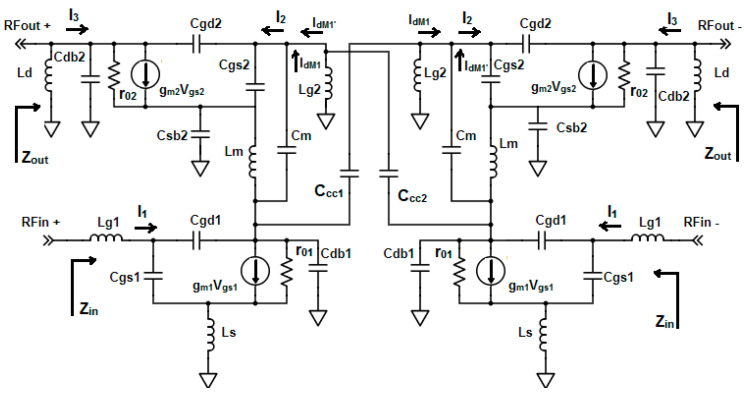
Small-signal equivalent circuit of proposed LNA.

Figure 2 depicts the small-signal equivalent model of the proposed differential LNA. This model serves as the basis for the study of gain, linearity, and power optimization. Additionally, when viewed as a two-port network, it provides insight into the input and output impedance characteristics.

#### 2.1.1. Gain Improvement Using Current-Reuse Technique

**Figure 3:**
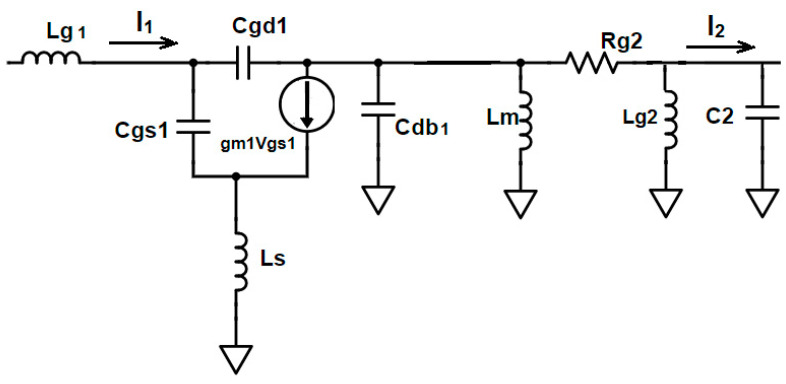
Small-signal equivalent of input CS stage.

Gain improvement is assessed by analyzing the overall current gain of the proposed LNA. This gain is determined using the small-signal equivalent circuit illustrated in Figure 3.

The overall current gain (A_I_) of the proposed differential LNA can be calculated from the current gain of M_1_ (I_2_/I_1_) and M_2_ (I_3_/I_2_):

(1) $$A_{I}=\frac{I_{2}}{I_{1}}*\frac{I_{3}}{I_{2}}$$

The MOS transistors (M_1_ and M_2_) are operated in the saturation region with a large voltage headroom to achieve a high gain at the cost of power dissipation. Here, the current-reuse technique is used to boost the current gain of the proposed LNA with low power via the presence of ‘C_m_’.

In Figure 3, the current gain of CS transistor M_1_ is given by

(2) $$\frac{I_{2}}{I_{1}}=\frac{g_{m1}}{s(C_{gs1}+C_{gd1})}\frac{\left(((sL_{m\;}||\;\frac{1}{{sC}_{db1}})+R_{g2})\;||\;sL_{g2\;}\right)}{\left(((sL_{m\;}||\;\frac{1}{{sC}_{db1}})+R_{g2})\;||\;sL_{g2\;})+(\frac{1}{sC_{2}}\right)}$$

where

L_m_ is the interstage matching inductor; L_g2_ is the gate inductor of M_2_;

C_db1_ is the drain-to-bulk capacitance of M_1_;

R_g2_ is the gate resistance of M_2_;

C_2_ = $C_{m}+C_{gs2}+C_{cc2}$.

**Figure 4:**
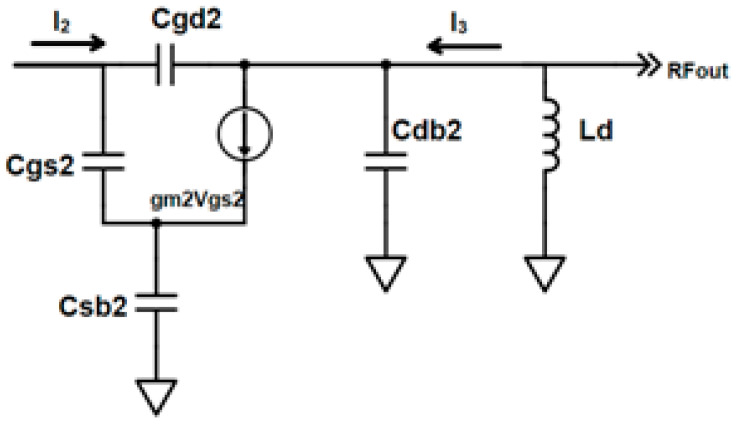
Small-signal equivalent of cascode stage.

This is the equivalent capacitance composed of interstage capacitance C_m_, the gate-to-source capacitance of M_2_, C_gs2_, and cross-coupling capacitor C_CC2_ obtained from the small-signal equivalent circuit shown in Figure 2 and Figure 4.

In Figure 4, The current gain of cascode transistor M_2_ is given by

(3) $$\frac{I_{3}}{I_{2}}=\frac{2C_{cc2}}{C_{gs2}+C_{gd2}+C_{cc2}}g_{m2}\frac{sL_{d\;}}{\left(sL_{d\;}||\frac{1}{{sC}_{db2}}\right)}$$

The current gain (A_I_) of the differential cascode LNA with g_m_ boosting is achieved from the individual current gain of CS and cascode LNA.

On substituting Equation (2) and (3) in Equation (1), the current gain is

(4) $$A_{I}=\frac{I_{3}}{I_{1}}=\left[\frac{g_{m1}}{s(C_{gs1}+C_{gd1})}\frac{\left(((sL_{m\;}||\;\frac{1}{{sC}_{db1}})+R_{g2})\;||\;sL_{g2\;}\right)}{\left(((sL_{m\;}||\;\frac{1}{{sC}_{db1}})+R_{g2})\;||\;sL_{g2\;})+(\frac{1}{sC_{2}}\right)}\right]*\left[\frac{2C_{cc2}}{C_{gs2}+C_{gd2}+C_{cc2}}g_{m2}\frac{sL_{d\;}}{\left(sL_{d\;}||\;\frac{1}{{sC}_{db2}}\right)}\right]$$

From the current gain expression of A_I_ in Equation (4), it is clear that the gain is boosted twice by the presence of the product of transconductances (g_m1_ andg_m2_). Thus, the current gain is improved using the current-reuse capacitor (C_m_) under the same DC current with low power.

#### 2.1.2. LNA Linearity Improvement Using Capacitor Cross-Coupling Technique

The CCC technique [35] enhances the linearity of LNA while reducing the gate–drain capacitance of the input common-source (CS) transistor, M_1_. To achieve a higher IIP3 (third-order input intercept point), it is imperative to minimize g_m3_, the nonlinear coefficient of M_1_.

In our proposed approach, to enhance IIP_3_, we utilize cross-coupling capacitors, C_CC1_ and C_CC2_, as illustrated in Figure 1, to mitigate the nonlinear effect of g_m3_. The gate inductor, L_g2_, which is connected to the gate of the cascode transistor, resonates with the cross-coupled capacitors, effectively eliminating the nonlinearity and noise contribution of the cascode stage.

The impact of CCC, which improves the linearity, can be studied using a variation inthe drain current I_d_ of the MOS device given by the Taylor series as

*I_d_* = *g_m1_v_gs_* + *g_m2_v_gs_^2^* + *g_m3_v_gs_^3^* (5)

where

g_m1_ is the main transconductance of the MOSFET;

g_m2_ is the second-order nonlinear coefficient;

g_m3_ is the third-order nonlinear coefficient.

The coefficients g_m1_, g_m2_, and g_m3_ are given by

(6) $$g_{m1}=\frac{\partial i_{d}}{\partial v_{gs}};\;g_{m2}=\frac{\partial^{2}i_{d}}{\partial v_{gs}^{2}};\;g_{m3}=\frac{\partial^{3}i_{d}}{\partial v_{gs}^{3}}$$

From the above given Equation (6), it is evident that the MOS device exhibits nonlinear behavior.

The IIP_3_ of the nonlinear device is given by

(7) $${\mathrm{IIP}}_{3}=\sqrt{\frac{4}{3}\left|\frac{g_{m1}}{g_{m3}}\right|}$$

From Equation (5), the drain currents flowing through M_1_ and M_1′_ are given by

*I_dM1_* = *g_m1M1_v_gsM1_* + *g_m2M1_v_gsM_^2^* + *g_m3M1_v_gsM1_^3^* (8)

*I_dM1_*_′_ = *g_m1M1′_v_gsM2_* + *g_m2M1′_v_gsM2_^2^* + *g_m3M1′_v_gsM2_^3^* (9)

where *V_gsM2_* is the gate-to-source voltage of M_2_ given by

*V_gsM2_* = *a_1_v_gsM1_* + *a_2_^2^ v_gsM1_^2^* + *a_3_^2^ v_gsM1_^3^* (10)

The resulting current, I_2_,is given by

*I_2_* = *I_dM1_* + *I_dM1′_* (11)

≈(*g_m1M1_* + *a_1_ g_m1M1′_*) *v_gsM1_* + (*g_m2M1_* + *a_1_^2^ g_m2M1′_*) *v_gsM1_^2^* + (*g_m3M1_* + *a_1_^3^ g_m3M1′_*) *v_gsM1_^3^* (12)

*I_2_* ≈ *g_m1M1_* + *a_1_ g_m1M1′_* (13)

In Equation (12), it is important to note that g_m3_M_1_ and g_m3_M_1′_ are negative when operating in the strong inversion region for NMOS transistors. Coefficient a_1_, representing the impedance when looking at the gate of M_2_, is a frequency-dependent parameter and is inherently negative according to basic circuit theory [36]. The coefficient of the third term in Equation (12) is offset by adjusting the gate bias of M_2_ and by resonating the gate inductor L_g2_ with the cross-coupling capacitors (C_CC1_ and C_CC2_).

Consequently, the resulting current, as given in Equation (13), demonstrates that the nonlinearity components related to g_m3_ are effectively canceled at M_1_, allowing the primary transconductance to be directed toward M_2_.

#### 2.1.3. Transconductance Improvement with Low-Power Body Biasing Technique

Typically, the body of an MOS transistor operates in the weak inversion region. Through the utilization of the forward body biasing technique, the MOS transistor’s body is biased to transition into the strong inversion region. This transition effectively reduces the threshold voltage (VTH) of the device, subsequently leading to a decrease in power consumption. This reduction in the threshold voltage significantly enhances the transconductance (gm), as demonstrated in Equation (14). The following equations describe the variation in g_m_ with V_TH_:

(14) $$\frac{\partial g_{m}}{\partial V_{TH}}\;\;\frac{I_{d0}}{2{\left(nU_{T}\right)}^{2}}\left(e^{\left(V_{GS}-\frac{V_{TH}}{2nU_{T}}\right)}\right)$$

where

(15) $$V_{\mathrm{TH}}=V_{\mathrm{TH}0}+\gamma (\sqrt{\left|2\Phi_{F}-V_{BS}\right|}-\sqrt{\left|2\Phi_{F}\right|})$$

V_TH0_ is the threshold voltage without the bulk-source voltage.V_BS_ = 0.

*Γ* is a process-dependent body effect parameter.

$\Phi_{F}$ is the substrate Fermi potential with typical values of 0.3*−*0.4V^1/2^. 

n is the substrate factor, whose value depends on the process and varies from 1 to 2.

U_T_ is defined as kT/q, the thermal voltage.

As inferred from the above Equation (14), g_m_ varies exponentially with respect to V_TH_. Biasing the body of the M_2_ transistor reduces the threshold voltage and increases transconductance g_m2_. With the increase in gm2, gain is also proportionally enhanced, as discussed in Equation (4), with a low power consumption.

### 2.2. Impedance Calculation at Input and Output

Using the small-signal equivalent circuit shown in Figure 2, the input impedance of the proposed differential LNA is given by

(16) $$Z_{\mathrm{in}}=s(L_{g1}+L_{s})+\frac{1}{{sC}_{gs1}}+\omega_{T}\;L_{s}$$

where

(17) $$\omega_{T}=\frac{g_{m1}}{C_{gs1}}$$

g_m1_ is the transconductance of M_1_; C_gs1_ is the parasitic gate-to-source capacitance of gainM_1_; and L_g1_ and L_s_ are the gate and source inductances.

Input matching is achieved by equating the real part of Equation (16) to source impedance (Rs = 50 Ω) and the imaginary part to zero to obtain the values of the source and gate inductors.

(18) $$L_{s}=\frac{R_{s}C_{gs1}}{g_{m1}}$$

(19) $$L_{s}+L_{g1}=\frac{1}{\omega_{0}^{2}C_{gs1}}$$

(20) $$C_{gs1}=\frac{1}{\omega_{0}^{2}(L_{g1}+L_{s})}$$

The output frequency (f_0_) of the proposed LNA can be calculated using

(21) $$f_{0}=\frac{1}{\sqrt{C_{gs1}(L_{g1}+L_{s})}}$$

The impedance seen at the output is given by

(22) $$Z_{\mathrm{out}}={\mathrm{sL}}_{d}||\;\frac{1}{{sC}_{db2}}$$

where

L_d_ is the drain inductor;

C_db2_ is the drain-to-bulk capacitance of M_2_.

## 3. Development of ANN Model

Neural networks are information processing systems designed with inspiration from the cognitive capabilities of the human brain. These networks abstractly generalize and learn from data, drawing upon their capacity to interpret patterns. An ANN model is capable of estimating various amplifier parameters based on simulation results, offering an alternative approach to traditional simulation tools.

### Neural Network Model for the Proposed CMOS Differential LNA

**Figure 5:**
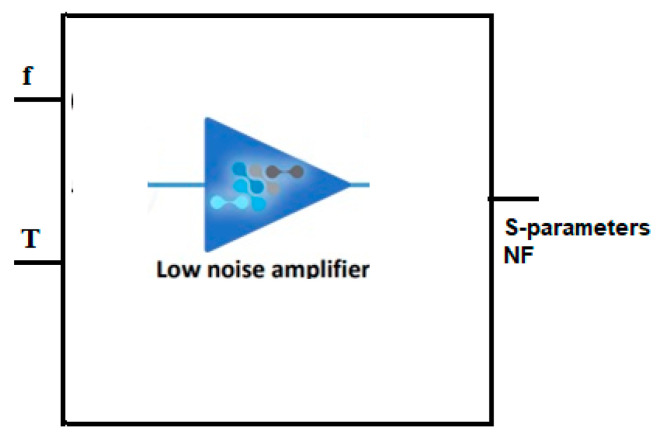
Parameters considered for neural network modeling of the proposed LNA.

The proposed LNA is meticulously designed, incorporating current-reuse, body biasing, and capacitor coupling techniques to achieve optimized gain, a low power consumption, improved linearity, and reduced noise. The key performance parameters, including S-parameters and the noise figure (NF), exhibit proportional variations with temperature across different frequencies. Consequently, both temperature and frequency are considered as input variables. The output parameters, S_21_ and NF, serve as the output variables in the modeling of the proposed LNA, as depicted in Figure 5.

**Figure 6:**
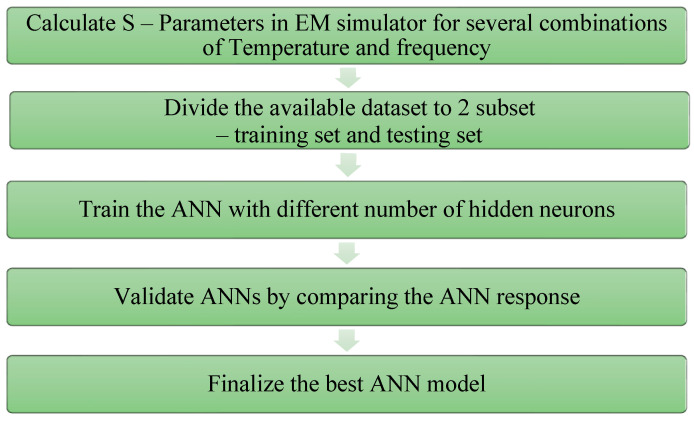
Flowchart of ANN modeling of the proposed LNA.

A flowchart for the modeling of the LNA using ANN is illustrated in Figure 6. The proposed LNA is simulated through Cadence, and performance parameters are obtained for various combinations of temperature and frequency. For each set of circuit parameters, the S-parameters and noise figure (NF) are determined, and the corresponding input dataset is generated.In line with the Pareto principle, the developed dataset is partitioned, with 80% allocated for training and the remaining 20% for testing and validation purposes. The ANN is then trained using different numbers of hidden neurons across various neural networks and algorithms. The model’s performance is subsequently validated based on the accuracy achieved through the best test statistics.

**Figure 7:**
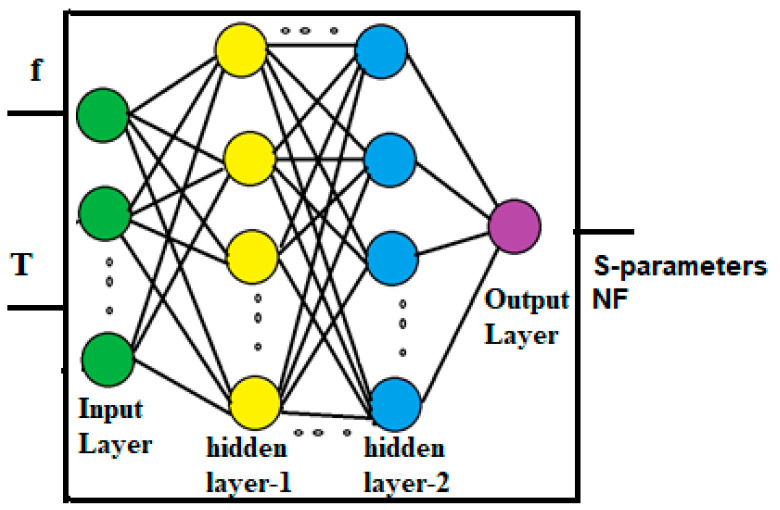
Neural network model developed for the proposed LNA.

An MLPNN model is developed for the proposed LNA, as shown in Figure 7, for the S-parameters and noise figure (NF).

Each ANN is equipped with input neurons that correspond to specific circuit parameters, as well as an output neuron that corresponds to the parameter being modeled. Once the ANN is trained, the desired parameter can be readily calculated based on its response. The model’s accuracy is validated by comparing the ANN’s response with the simulation results.

Specialized versions of feedforward networks, such as PatternNet, FitNet, and Cascade ForwardNet, are available and suitable for various types of input-to-output mapping. Consequently, these networks are chosen to explore the different ANN types while examining the impact of varying the number of hidden layers (NHL) on output accuracy. These neural networks are trained using various algorithms, as listed in Table 1.

Bayesian regularization is proposed for modeling this LNA. Typically, the dataset is partitioned into three subsets: training, testing, and validation. The training set error steadily decreases as the training progresses, while the validation set error initially reaches a minimum and then increases as the training continues. This phenomenon can lead to a less predictive model, and it is commonly referred to as overtraining. To overcome this issue, Bayes’ theorem is incorporated into the regularization scheme.

The cost function or sum squared error in the data to be minimized is given as

(23) $$S(w)=\beta \sum_{i=1}^{N_{D}}{\left[Y_{i}-f(X_{i})\right]}^{2}+\alpha \sum_{j=1}^{N_{w}}w_{j}^{2}$$

where

N_d_ is the number of rows in the input vector X;

N_w_ is the number of weights;

X_i_ is the input vector;

Y_i_ is the output vector.

Given the initial values of hyperparameters α and β, the cost function S(w) is minimized with respect to weights w.

## 4. Results and Discussion

**Figure 8:**
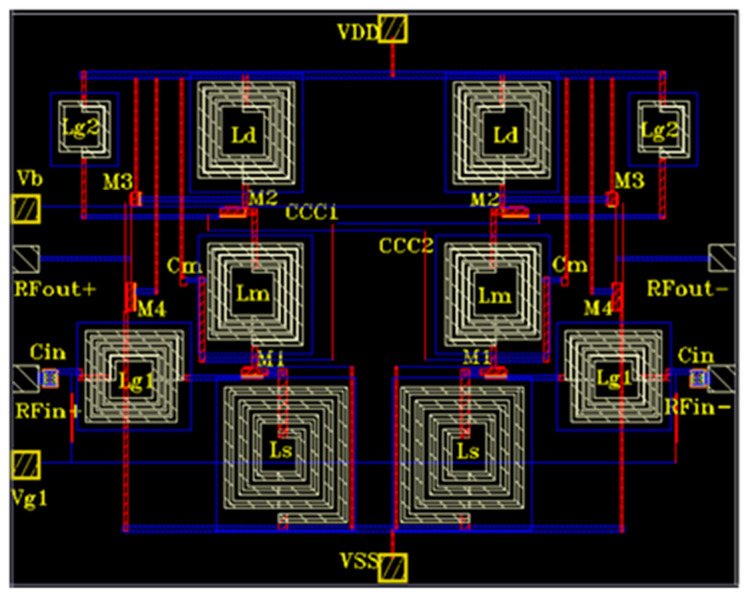
Layout of proposed differential LNA.

The proposed differential LNA was designed and simulated using 180 nm CMOS technology, operating at a frequency of 5 GHz, as depicted in Figure 8. The simulation was carried out within the Cadence Spectre environment. Square spiral inductors and capacitors from the technology library available in Cadence Spectre were utilized. For signal lines, Metal 6 was employed to ensure high conductivity, while input and output lines were implemented using Metal 4 to achieve good impedance matching. Intermediate connections were established using Metal 1 and Metal 2. To enhance signal transmission, the gain path of the proposed LNA was widened to reduce resistance effectively. The component values for the proposed LNA are provided in Table 2. The physical footprint of the proposed LNA occupies a silicon area measuring 0.8 × 0.6 mm^2^. A process corner analysis was conducted at various temperatures:Typical-Typical (TT, 27 °C), Fast-Fast (FF, 0 °C), and Slow-Slow (SS, 80 °C). 

### 4.1. Effect of Body Biasing and Current-Reuse Technique on LNA Gain

**Figure 9:**
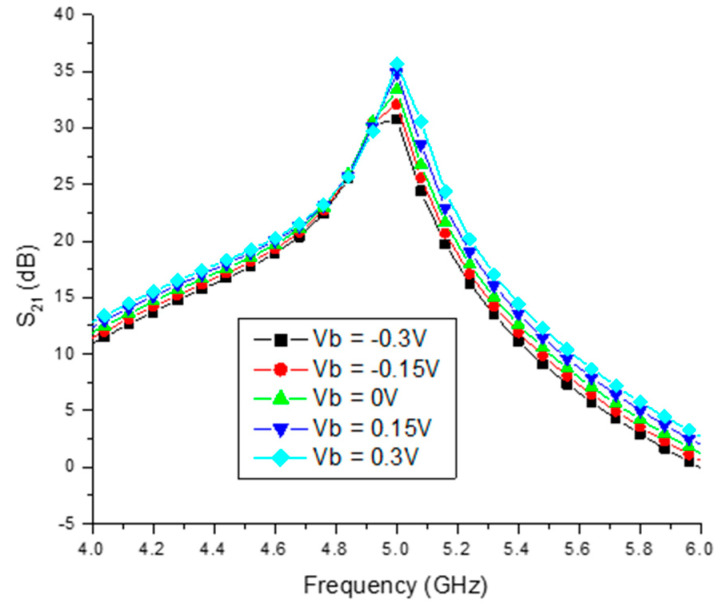
Body bias (V_b_) vs. gain (S_21_) of differential LNA.

Figure 9 illustrates the gain improvement (S21) as the bulk voltage (Vb) is varied from −0.3 V to 0.3 V. Notably, it is observed that the gain of the proposed LNA reaches 29.5 dB at the bulk voltage of Vb = −0.3 V. Furthermore, it can be inferred that the gain within the frequency range of 4.9 GHz to 5 GHz measures 29 dB, highlighting its exceptional performance in comparison to previously reported designs [6,8].

**Figure 10:**
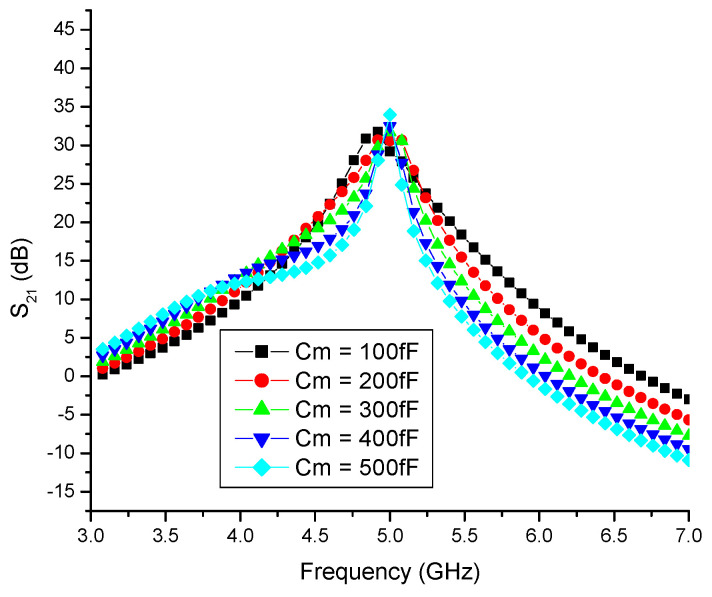
Current-reuse capacitor (C_m_) vs. gain (S_21_)of differential LNA.

The current-reuse (CR) technique is applied using the middle capacitor, C_m_. As C_m_ is varied from 100 fF to 500 fF, the gain demonstrates an improvement, increasing from 28.8 dB to 33.2 dB, as depicted in Figure 10. It is evident in the figure that the maximum gain is achieved when C_m_ equals 200 fF at the desired frequency of 5 GHz. Consequently, the gain is enhanced twofold through the combined use of the body biasing (BB) and CR techniques, in accordance with Equation (14).

### 4.2. Effect of Capacitor Cross-Coupling on LNA Linearity

**Figure 11:**
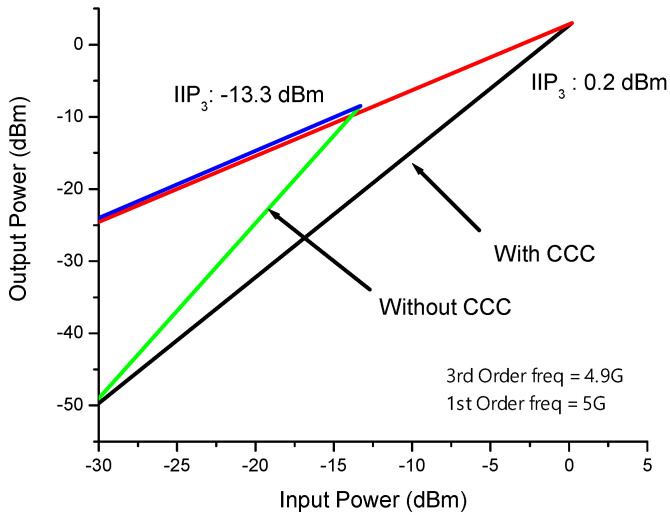
IIP3 of proposed LNA.

The linearity of the LNA is assessed through the third-order input intercept point (IIP3). In receiver LNAs, it is expected that the IIP3 should exceed −10 dBm. The IIP3 of the proposed LNA is determined through simulation using a two-tone signal with a 100 MHz spacing. As depicted in Figure 11, the proposed LNA achieves an IIP3 of 0.2 dBm with capacitor cross-coupling and −13.3 dBm without capacitor cross-coupling. Consequently, the utilization of the CCC technique results in a significant improvement of 13 dBm.

### 4.3. Simulation Results at Different Process Corners

**Figure 12:**
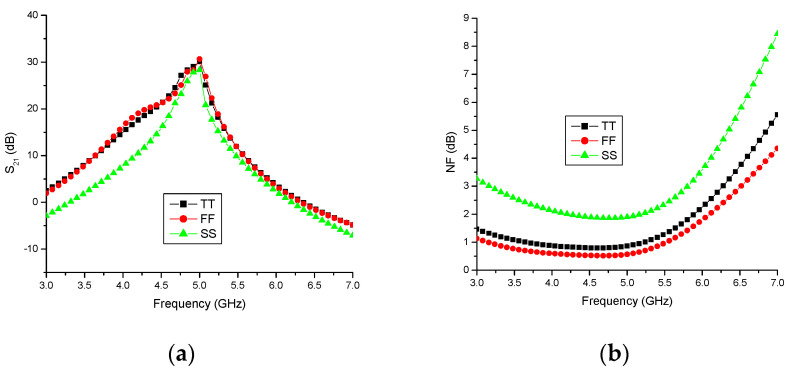

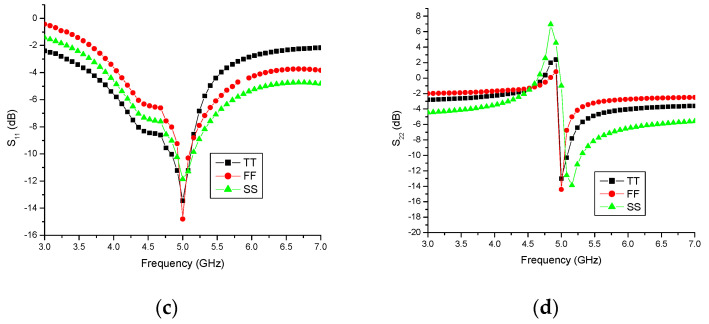
Simulation results at different process corners: (**a**) gain (S21), dB; (**b**) noise figure (NF), dB; (**c**) input return loss (S11), dB; and (**d**) output return loss (S22), dB.

Figure 12 presents the simulation results of the differential LNA under different process corners. Notably, it is observed that the proposed differential LNA delivers superior performance at the Fast corner (0 °C) when compared to the Typical (27 °C) and Slow corners (80 °C). The gain of the proposed LNA under different corners is illustrated in Figure 12a. It is evident that the gain exceeds 25 dB within the operational bandwidth (4.8–5.2 GHz) and reaches 30 dB at the desired frequency. Furthermore, it is noteworthy that the gain remains consistent between the Typical and Fast corners.

The noise figure (NF) of the LNA must be below 3 dB within the desired frequency range. Figure 12b depicts the NF at various corners, revealing an NF of 1.2 dB at the Typical corner, 0.7 dB at the Fast corner, and 1.95 dB at the Slow corner for the desired frequency. These results signify that the noise performance is excellent at the Fast corner.

In terms of the input return loss (S_11_), it is imperative that it remains below −10 dB at the required frequency of operation. Figure 12c shows that S_11_ measures−13.3 dB at the Typical corner, −14.5 dB at the Fast corner, and −12.1 dB at the Slow corner within the desired frequency range. This indicates that S11 is particularly strong at the Fast corner when compared to the SS and TT corners.

Similarly, the output return loss (S_22_) should also be less than −10 dB at the required frequency of operation. As illustrated in Figure 12d, S_22_ is −13.1 dB at the Typical corner, −14.8 dB at the Fast corner, and −11.3 dB at the Slow corner within the desired frequency range. Additionally, the results suggest that the output return loss is nearly consistent between the Typical and Fast corners.

The performance parameters such as gain, NF, input and output return loss, and power dissipation at various process corners are analyzed and tabulated in Table 3. 

### 4.4. Performance Comparison of Proposed LNA with Existing State of Art 

The performance of the proposed LNA is compared to that of several previously reported differential LNAs designed for wireless applications, and the results are summarized in Table 4. The proposed LNA achieves a gain of 29.5 dB, a noise figure of 1.2 dB, and an IIP3 of 0.2 dBm, all while operating at a reduced supply voltage of 0.9 V. These results outperform the reported works [6,7,8].

It is noteworthy that the proposed LNA exhibits a Figure of Merit (FoM) of 24.26, which is notably higher than in all the other reported works. The advantages brought by the proposed design techniques, such as the body-biased differential architecture combined with current reuse and capacitor cross-coupling, are evident, especially in achieving a highly linear and low-power solution. 

The Figure of Merit (FoM) is calculated using Equation (24):

(24) $$\mathrm{FoM}=\frac{Gain\left(abs\right)*{IIP}_{3}\left(mW\right)}{\left(NF\right)\left(abs\right)*P_{dc}\left(mW\right)}$$

### 4.5. Performance Comparison with Different NN Models

This section can be divided into two parts. Figure 10 illustrates the impact of temperature on the S-parameters and noise figure (NF) for the frequency obtained from the Cadence simulations. ANN models were constructed for the CMOS differential LNA with 2 input neurons, 25 hidden neurons, and 2 output neurons. These models were trained using 750 datasets, employing 13 different neural network algorithms to identify high efficiency and performance rates. The performance rate varied across the different neural networks, and the best-performing neural network was subsequently analyzed.

Table 5 presents a performance comparison of the various neural networks developed for the proposed CMOS differential LNA. ANN training was carried out using different neural networks while keeping the number of hidden layers constant. It was observed that PatternNet, FitNet, and CascadeForwardNet consistently achieved accuracy levels exceeding 99% when utilizing 25 hidden neurons in the BR algorithm.

Table 6 displays the Mean Relative Error (MRE) and Root Mean Square Error (RMSE) for various algorithms implemented with PatternNet. Based on the results obtained, the PatternNet-BR algorithm exhibits a lower MRE, making it the preferred choice for our LNA modeling.

Table 7 presents the accuracy achieved using the BR algorithm with different neural networks and various numbers of hidden layers, including 5, 10, 25, and 30 neurons. The results indicate that an accuracy of over 99% was consistently achieved with the neural networks with 25 to 30 neurons for the modeling of this LNA.

### 4.6. Performance Comparison between Cadence Simulation and Developed ANN Results

To account for temperature variations ranging from 0 °C to 80 °C, the LNA parameters are compared between the results from the Cadence simulations and those from ANN.

Table 8 and Table 9 provide sample training and testing data, along with the results obtained from the Cadence simulations and ANN modeling. These tables illustrate that the S-parameters and NF values for the proposed LNA are nearly identical to the results obtained through the ANN modeling. Each sample number corresponds to a specific frequency (in GHz) for a given temperature (in degrees).

**Figure 13:**
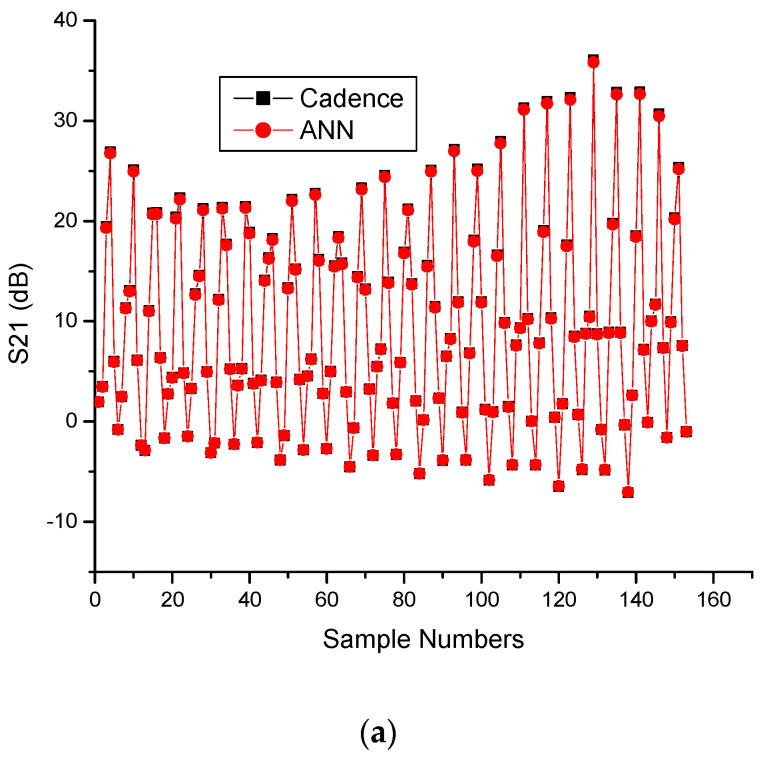

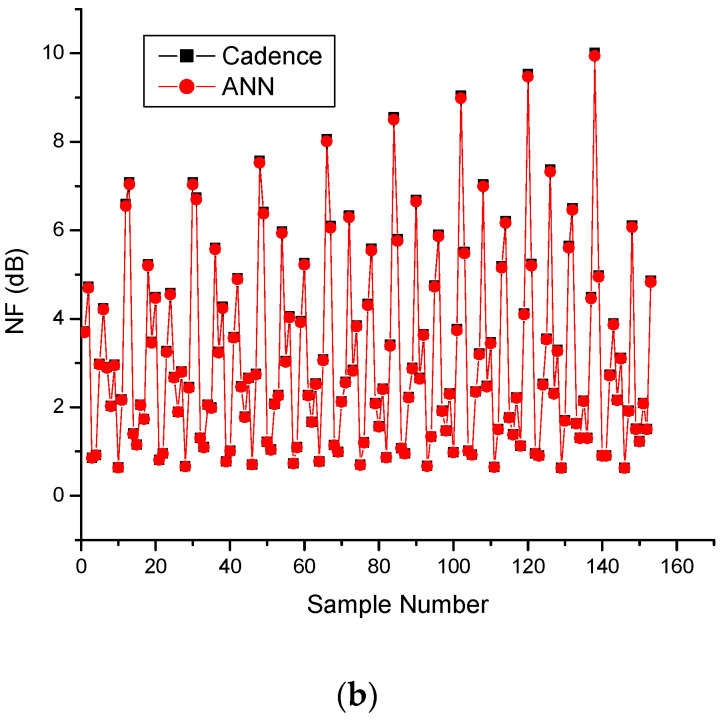
CMOS differential LNA results obtained using Cadence and ANN: (**a**) S21, (**b**) NF.

Figure 13 presents a comparison between the Cadence simulations and the results generated through the ANN modeling for the CMOS differential LNA parameters. The observation indicates that the results for the proposed LNA are almost identical.

## 5. Performance Comparison with the Existing State of Art

Table 10 provides a performance comparison with the existing state of the art. It is evident that the proposed LNAs are modeled using the MLPNN–BR algorithm, which proves to be more robust than the standard back-propagation method. This algorithm also demonstrates the ability to uncover potentially complex relationships, even when the data are limited; requires less formal statistical training; reduces or eliminates the need for extensive cross-validation; and prevents over-fitting. This comparison is made against the reported works [37,38,44,45,46].

## 6. Conclusions

This paper introduces Artificial Neural Network (ANN) modeling of a proposed LNA, which is a CMOS differential LNA designed to enhance gain, linearity, and power efficiency. The adaptation of the capacitor cross-coupling technique involves the use of cross-coupling capacitors alongside the gate inductor of the cascode device to resonate the nonlinear component found in the CS stage while allowing the main transconductance to reach the output. Gain is increased through the implementation of the CR and BB techniques, all while maintaining a low power consumption. The proposed LNA achieves remarkable results with a 29.5 dB gain, a 1.2 dB NF, and an IIP3 of 0.2 dBm, all with a reduced supply voltage of 0.9 V.

It is evident that this LNA exhibits a high gain, a low NF, and excellent linearity at the desired frequency, even with a reduced supply voltage. The modeling of the proposed LNA using PatternNet BR reveals that the simulation results closely match those of the developed ANN. When compared with the Cadence simulation method, the proposed technique can also provide precise circuit solutions. While simulation or measurement data may be used for modeling, the proposed method’s advantages are more pronounced, especially in cases where equivalent circuits for simulation are unavailable or when measurements are costly.

## Figures and Tables

**Table 1:** Different neural network algorithms.

S. No	Algorithm	Abbreviation
1	scg	Scaled conjugate gradient back propagation
2	cgb	Conjugate gradient back propagation with Powell/Beale restarts
3	bfg	BFGS quasi-Newton back propagation
4	cgp	Conjugate gradient back propagation with Polak–Ribiére updates
5	gda	Gradient descent with adaptive learning rate back propagation
6	gd	Gradient descent back propagation
7	gdm	Gradient descent with momentum back propagation
8	gdx	Gradient descent with momentum and adaptive learning rate back propagation
9	lm	Levenberg–Marquardt back propagation
10	cgf	Conjugate gradient back propagation with Fletcher–Reeves updates
11	oss	One-step secant back propagation
12	rp	Resilient back propagation
13	Br	Bayesian regularization

**Table 2:** Component values of the proposed LNA.

Component	M1	M2	Ls	Lg1	Lg2	Lm	Cin	Cm	Ccc1	Ccc2
Values	138 µm	216 µm	1.15 nH	2 nH	0.05 nH	2.29 nH	1.63 pF	0.2 pF	0.1 pF	0.1 pF

**Table 3:** Performance of the proposed LNA in normal, best, and worst cases at 5 GHz.

Parameter	TT 27 °C	FF 0 °C	SS 80 °C
S_11_ (dB)	−13.3	−14.5	−12.1
S_22_ (dB)	−13.1	−14.8	−11.3
S_21_ (dB)	29.5	30.6	28.4
NF (dB)	1.2	0.7	1.9
P_dc_ (mW)	19.3	17.2	21.3

**Table 4:** Performance summary with existing state of the art LNAs.

State of the Art	Freq (GHz)	Tech (µm)	S_21_ (dB)	NF (dB)	V_dd_ (V)	P_dc_ (mW)	IIP_3_ (dBm)	FoM	Remarks
[6]	5.8	0.18	18.66	2.03	1.8	7.58	-	6.07	Low gain, high NF
[7]	2.4	0.18	20.285	1	1.8	167.1	-	0.51	High power consumption
[8]	5.5	0.18	16.5	1.53	0.5	0.89	−17.2	0.67	Low gain, low IIP3
This work	5	0.18	29.5 *	1.2	0.9	19.3	0.2	24.26	High gain, low NF, and better linearity

* with buffer.

**Table 5:** Percentage accuracy obtained with different neural networks for the proposed LNA.

Algorithm	PatternNet	FitNet	Cascade ForwardNet
SCG	97.33	98	97.33
CGB	76	97	98
BFG	27	35	40
CGP	99.1	96	92.6
GDA	26.67	8.6	2
GDA	2	4	4
GDM	5.3	4	1.3
GDX	58.6	34	25.3
LM	3.3	4	5
CGF	82.6	86.67	93.33
OSS	29	77.3	86.67
RP	66.7	69.3	77.33
BR	99.34	98	99

**Table 6:** MRE and RMSE for different algorithms of PatternNet.

Algorithm	Accuracy(%)	MRE	RMSE
SCG	97.33	2.67	1.63
CGB	76	24	4.90
BFG	27	73	8.54
CGP	99.1	0.9	0.95
GDA	26.67	73.33	8.56
GDA	2	98	9.90
GDM	5.3	94.7	9.73
GDX	58.6	41.4	6.43
LM	3.3	96.7	9.83
CGF	82.6	17.4	4.17
OSS	29	71	8.43
RP	66.7	33.3	5.77
BR	99.34	0.66	0.81

**Table 7:** Accuracy obtained for BR algorithm with different numbers of hidden neurons.

Hidden Neurons	S21	NF
5	83.57	86.30
10	90.23	92.71
20	97.14	96.42
25–30	99.97	99.79

**Table 8:** Sample training data and the results obtained from Cadence and ANN.

Sample No.	Cadence—S_21_ (Real Values)	ANN—S_21_ (Predicted Values)	MRE	RMSE	Cadence—NF (Real Values)	ANN—NF (Predicted Values)	MRE	RMSE
1	−18.1001	−17.9806	−0.1195	0.345688	3.71105	3.68656	0.02449	0.156493
2	−7.32325	−7.27491	−0.04834	0.219864	4.72765	4.69645	0.0312	0.176635
3	13.907	13.81521	0.09179	0.302969	0.85553	0.84988	0.00565	0.075166
4	31.16381	30.95813	0.20568	0.45352	0.92032	0.91425	0.00607	0.07791
5	10.4285	10.35967	0.06883	0.262355	2.97907	2.95941	0.01966	0.140214
6	8.28715	8.23246	0.05469	0.233859	4.23488	4.20693	0.02795	0.167183
7	−6.22354	−6.18246	−0.04108	0.202682	2.9065	2.88731	0.01919	0.138528
8	−7.25494	−7.20706	−0.04788	0.218815	2.0291	2.01571	0.01339	0.115715
9	4.95072	4.91804	0.03268	0.180776	2.96014	2.94061	0.01953	0.13975
10	34.69036	34.46141	0.22895	0.478487	0.63707	0.63287	0.0042	0.064807
11	8.71366	8.65615	0.05751	0.239812	2.17553	2.16117	0.01436	0.119833
12	0.79462	0.78938	0.00524	0.072388	6.59107	6.54757	0.0435	0.208567
13	−16.9761	−16.864	−0.1121	0.334813	7.08496	7.0382	0.04676	0.216241
14	2.83459	2.81588	0.01871	0.136785	1.40329	1.39403	0.00926	0.096229
15	5.60129	5.56432	0.03697	0.192276	1.1591	1.15145	0.00765	0.087464
16	31.96165	31.7507	0.21095	0.459293	2.06107	2.04747	0.0136	0.116619
17	16.14315	16.03661	0.10654	0.326405	1.73748	1.72601	0.01147	0.107098
18	−0.88958	−0.88371	−0.00587	0.076616	5.23356	5.19902	0.03454	0.185849
19	−16.8985	−16.787	−0.1115	0.333916	3.47562	3.45268	0.02294	0.15146
20	−6.08266	−6.04252	−0.04014	0.20035	4.49132	4.46167	0.02965	0.172192
21	15.62884	15.52568	0.10316	0.321185	0.81078	0.80543	0.00535	0.073144
22	33.539	33.31764	0.22136	0.470489	0.95501	0.9487	0.00631	0.079436
23	9.0474	8.98769	0.05971	0.244356	3.26576	3.24421	0.02155	0.146799
24	7.52286	7.47321	0.04965	0.222823	4.57863	4.54841	0.03022	0.173839
25	−5.25111	−5.21646	−0.03465	0.186145	2.68156	2.66386	0.0177	0.133041

**Table 9:** Sample testing data and the results obtained from Cadence and ANN.

Sample No.	Cadence—S_21_ (Real Values)	ANN—S_21_ (Predicted Values)	MRE	RMSE	Cadence—NF (Real Values)	ANN—NF (Predicted Values)	MRE	RMSE
1	−5.99297	−5.95342	−0.03955	0.198872	1.89999	1.88745	0.01254	0.111982
2	6.57258	6.5292	0.04338	0.208279	2.80927	2.79073	0.01854	0.136162
3	32.24145	32.02865	0.2128	0.461303	0.663	0.65862	0.00438	0.066182
4	7.35635	7.30779	0.04856	0.220363	2.45318	2.43699	0.01619	0.12724
5	−0.01017	−0.0101	0.0001	0.008367	7.07881	7.03209	0.04672	0.216148
6	−15.7674	−15.6634	−0.104	0.32249	6.74143	6.69693	0.0445	0.21095
7	4.01011	3.98364	0.02647	0.162696	1.306	1.29738	0.00862	0.092844
8	7.37583	7.32715	0.04868	0.220635	1.09624	1.089	0.00724	0.085088
9	32.12838	31.91633	0.21205	0.460489	2.05571	2.04214	0.01357	0.11649
10	14.9347	14.83613	0.09857	0.313959	1.99324	1.98008	0.01316	0.114717

**Table 10:** Performance comparison with the existing state of the art.

State of the Art	Algorithm Used	Frequency	Parameters Considered		Error
			Input	Output	
[37]	M-MLPNN	4 GHz–6 GHz	12	5	--
[38]	MLPNN	1 GHz–4 GHz	-	2	0.005
[44]	MLPNN -LM	100 MHz–8 GHz	4	10	--
[45]	MLPNN-LM	300 MHz–18 GHz	4	10	--
[46]	Surrogate modeling	2 GHz–3 GHz	2	7	0.001
Proposed work	MLPNN-BR	5 GHz	2	2	0.001

## Data Availability

All data are included within manuscript.
